# Multiple omics analysis reveals that high fiber diets promote gluconeogenesis and inhibit glycolysis in muscle

**DOI:** 10.1186/s12864-020-07048-1

**Published:** 2020-09-24

**Authors:** Jianghong Wu, Ding Yang, Husile Gong, Yunxia Qi, Hailian Sun, Yongbin Liu, Yahong Liu, Xiao Qiu

**Affiliations:** 1 College of Animal Science and Technology, Inner Mongolia University for Nationalities, Tongliao, 028000 China; 2 Inner Mongolia Academy of Agricultural & Animal Husbandry Sciences, Hohhot, 010031 China; 3 College of Life Science, Inner Mongolia University for Nationalities, Tongliao, 028000 China

**Keywords:** Glucose metabolism, Integrative analysis, Meat quality, Metabolome, Ruminal microbiome, Transcriptome

## Abstract

**Background:**

Meat quality is a complex trait affected by genotypic and environmental factors. In a previous study, it was found that feedstuffs have various effects on the growth rate and meat quality of lambs. However, the underlying mechanisms are still not entirely clear.

**Results:**

In this study, to investigate the mechanisms that impact meat quality in twin sheep fed either with high fiber low protein (HFLP) forage (*Ceratoides*) or low fiber high protein (LFHP) forage (alfalfa) diets, multi omics techniques were utilized for integration analysis based on the feed nutritional value and the sheep microbiome, transcriptome, metabolome, and fatty acid profile. Results showed that the production performance and the muscle components of lambs were significantly affected by feeds. The essential fatty acid (linoleic acid and arachidonic acid) content of the muscle, based on gas chromatography-mass spectrometry analysis, was increased when lambs were fed with HFLP. The microbes in the lambs’ rumen fed a HFLP diet were more diverse than those of the LFHP fed group. Besides, the ratio of Bacteroidetes and Firmicutes in the rumen of the sheep fed a LFHP diet was 2.6 times higher than that of the HFLP fed group. Transcriptome analysis of the muscle revealed that the genes related to glucose metabolic processes and fatty acid biosynthesis were significantly differentially expressed between the two groups. Potential cross talk was found between the sfour omics data layers, which helps to understand the mechanism by which feedstuffs affect meat quality of lambs.

**Conclusion:**

Feed systems may affect the epigenetic regulation of genes involved in the glucose metabolic pathway. HFLP feeds could induce gluconeogenesis to maintain glucose levels in blood, resulting in decreased fat content in muscle. The multiple omics analysis showed that the microbiota structure is significantly correlated with the metabolome and gene expression in muscle. This study laid a theoretical foundation for controlling the nutrient intake of sheep; it suggested that its fatty acid spectrum modifications and the removal of meat quality detrimental material could guide sheep feeding for functional mutton.

## Background

Sheep (*Ovis aries*) are important livestock that provide meat, milk, and fiber. Both genetic factors and diet composition can influence lamb quality [1]. While differences in the fatty acid composition of individual animals of the same breed have been observed [2], the basis for this variation remains to be delineated [3]. Like all ruminants, sheep have a specialized digestive organ, the rumen, that breaks down cellulose from plant materials into simpler carbohydrates. The sheep rumen encompasses a complex microbiota and serves as the primary site for microbial fermentation of ingested feed [4]. Rumen microbes are intermediate links between the diet and the nutrients that are absorbed by sheep. Plant materials are fermented by microbes in the rumen and converted to mycoproteins, carbohydrates, and lipids, which are direct sources of nutrition for ruminants [5]. The rumen microbiota is a dynamic system in which both the microbial diversity and community structure are shaped when exposed to different diet compositions [6, 7]. It is known that high roughage diet feed can improve microbial protein synthesis in the rumen [8]. High-grain feeding is also capable of altering bacterial microbiota composition, fermentation, and local inflammation [9]. In addition, feeding patterns may also influence the rumen microbial ecology system [10]. Noteworthy, the quality of mutton changes after moving from grazing or traditional rearing systems to a stall feeding system; therefore, it is urgent to clarify the molecular mechanism by which feed affects meat quality.

Alfalfa is the most prevalent forage legume to livestock for stall-feeding system in worldwide. *Ceratocarpus arborescens* (also known as *Ceratoides arborescens (Losinsk.) Tsien et C. G. Ma*) is a forage grass with a higher fiber content than that of alfalfa. It is widely distributed in the north of China, which is important for winter grazing in arid and semi-arid grasslands [11]. Therefore, these feeds were chosen to compare their effect on lamb quality and to study the regulation mechanisms underlying such effects.

Although the rumen microbiota is considered to play an important role in sheep physiology, the mechanisms by which diets affect ruminants’ physiology remain poorly understood. Multi omics is a powerful technology to understand the interactions between the genotype, the environment, and life in a concerted way, and illustrate these complex traits. Recent, research has revealed that the gut microbiota plays an essential role in host metabolism [12] and energy harvest [13]. Fat deposition processes of a host change, when the microbiota of obese mice is transplanted into lean mice. Moreover, molecular interactions have been reported between the metabolome of the gut microbiota and the human physiology [14, 15]. These studies have mostly focused on monogastric animals. Although a lot of research has deepen on the relationship between diet and microbiota composition in cattle rumen, it has also been shown that rumen microbial community composition varies with the host [10].

Here, it was hypothesized that forage species may have an effect on both the rumen microbiota and meat quality of sheep due to differences in fiber and protein content, and nutrient availability. In this study, to analyze the association between feeds and lamb quality, systems biology methods were utilized to integrate multi omics data, such as feed nutrition, microbiome, transcriptome, metabolome and fatty acid profile. This research will provide a foundational understanding of how feeds affect lamb quality.

## Results

### Growth performance and carcass traits

Results showed that the group fed with LFHP pellets had significantly higher weight than the HFLP group (Table 1). The average daily gain is 185.9 g per day for the LFHP group significantly higher than that of the HFLP group (89.8 g/day, *P* < 0.001). These carcass traits suggested that the LFHP group had better growth performance. The fat content of the muscle was also higher in the LFHP group.

**Table 1:** Carcass traits of twin sheep fed two different diets

Trait	HFLP	LFHP	*P* value^a^
Start weight (kg)	23.83 ± 1.44	23.53 ± 1.86	0.662
Slaughter weight (kg)	33.70 ± 3.97	43.98 ± 8.56	0.042
Carcass weight (kg)	14.43 ± 1.65	19.58 ± 3.31	0.043
Dressing %	42.83 ± 0.95	44.74 ± 2.47	0.243
Loin eye area (cm^2^)	17.75 ± 0.21	27.71 ± 6.67	0.056
Water holding capacity	17.58 ± 1.33	21.02 ± 2.12	0.136
Protein	20.00 ± 0.84	19.25 ± 1.04	0.266
Lipid	3.67 ± 1.71	8.37 ± 2.52	0.026
Moisture	74.32 ± 1.26	70.65 ± 1.94	0.025
Fiber	0.92 ± 0.31	1.01 ± 0.51	0.687

^a^ **A** paired *t*-test was used to determine the significance of the difference between the two groups

### Firmicutes/Bacteroidetes ratio in the rumen and microbiota of the HFLP fed group

**Fig. 1:**
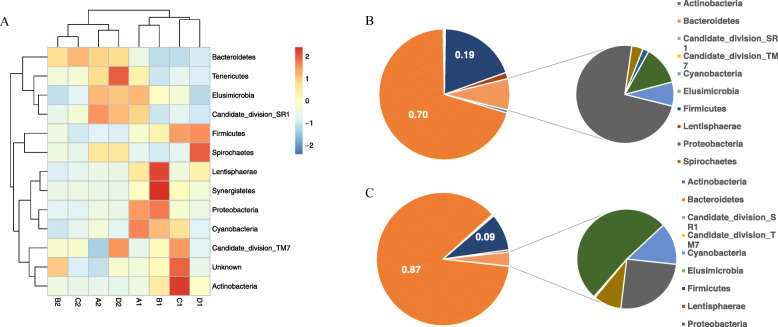
Changes in rumen microbiota of sheep raised under different feeds. **a**. hierarchical cluster based on the microbial content at the phylum-level. **b**. and **c**. Microbial content in the rumen of sheep fed with *Ceratoides* pellets (a HFLP diet) (B:F = 70.1:19.3 = 3.63) or; (**c**), alfalfa pellets (a LFHP diet) (B:F = 86.5:9 = 9.61)

To characterize the microbial community structure of the rumen of sheep groups fed with two different pellet feeds, we sequenced the 16S rRNA gene of rumen bacteria. A total of 1230 OTUs were identified among the eight rumen samples tested. Analysis of the relative abundance of bacterial OTUs revealed that the two diets had different impact on the rumen microbiota. The biodiversity of the rumen microbiota was higher in the *HFLP* fed group than that in the LFHP fed group, based on the alpha diversity indexes obtained using the Mothur software (Table 2). We hierarchically clustered bacterial phylotypes at phylum-level by the similarity of their dynamics across feeds and samples (Fig. 1a). Besides, results showed that the community microbiota of the eight individuals under study was divided into two classes, consistent with the feed group to which they belonged. Moreover, results indicated that the structure of the rumen microbiota changed significantly according to the feeds rather than the genetics of individuals. Notably, members of the phyla Bacteroidetes and Firmicutes, two of the leading sources of rumen microbiota, were hypothesized to be sensitive to feed intake. Results showed that the Bacteroidetes/Firmicutes ratio in rumen microbiota of the LFHP fed group was 2.6 times higher than that of the HFLP fed group (Fig. 1b and c. However, the structure of the proximal duodenum microbiota was the reversal, being Firmicutes the predominant phylum rather than Bacteroidetes (data not shown).

**Table 2:** Statistical results of alpha diversity in rumen microbiota

Sample_ ID	Sobs	Ace	Chao1	Shannon	Simpson
A1	1015.00	1085.01	1094.10	5.12	0.02
A2	932.00	997.04	1031.62	4.02	0.13
B1	1073.00	1117.13	1129.29	5.06	0.02
B2	928.00	1000.85	1031.89	4.21	0.05
C1	968.00	1034.45	1041.22	5.21	0.01
C2	952.00	1017.24	1032.67	4.24	0.06
D1	912.00	970.03	973.26	4.84	0.02
D2	908.00	954.23	973.27	4.46	0.03

Note: A1 –D1 represents the HFLP group and A2-D2 represents the LFHP group. Sobs (observed species) represents the number of OTU. Chao1 and ace indexes reflected community richness. Simpson and Shannon indexes reflected community diversity

**Fig. 2:**
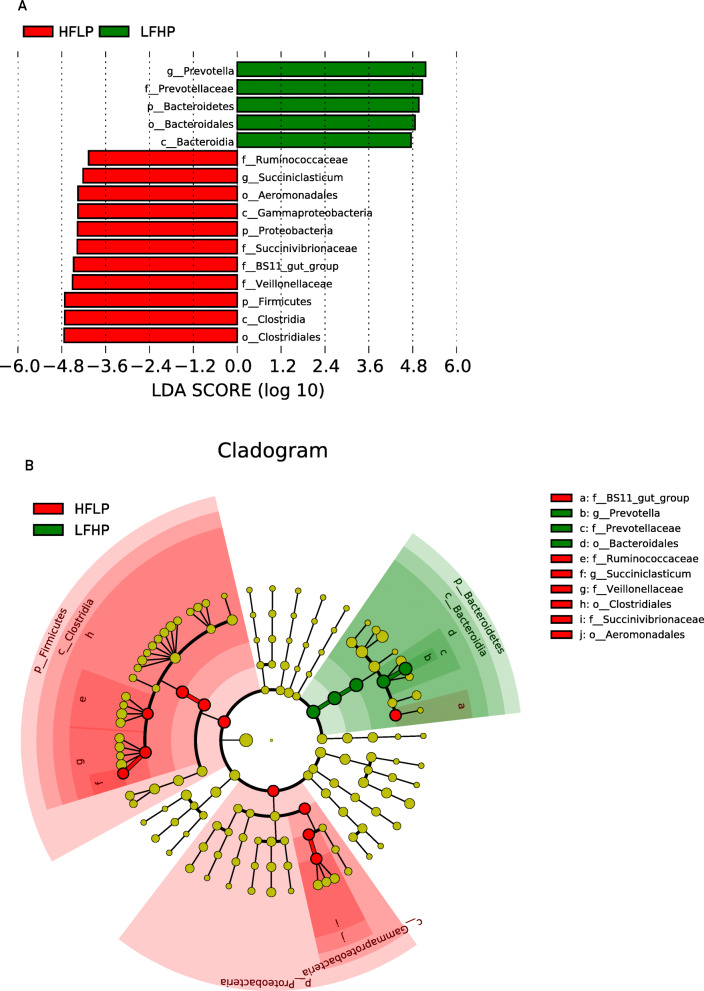
Differentially abundant taxa in the rumen of sheep feed different diets, based on LefSe analysis. **a**. Microbes, with significant abundance differences between the two sheep group, that have an LDA score greater than the estimated value. The length of the bar represents the log_10_ transformed LDA score. Green bars represent the taxa that were that were found to be more abundant in the LFHP, whereas red bars represent those that were more abundant in the HFLP group. **b**. Cladogram showing the microbial species with significant abundance differences between the two groups. Red and green shadows indicate different groups, with taxa classification at the level of phylum, class, order, family, and genus shown from the inside to the outside. The red and green nodes in the phylogenetic tree represent microbial species that play an important role in each of the two groups. Yellow nodes represent species with no significant difference

Enterotypes were also strongly associated with feeds, being the LFHP diet (alfalfa pellet) associated to Bacteroidetes predominance, and the HFLP diet (*Ceratoides* pellets) with that of Firmicutes. Thus, the LFHP group showed significant enrichment in Bacteroidetes and depletion in Firmicutes (*P* < 0.001), being bacteria from the genera *Prevotella* and *Succiniclasticum*, especially abundant. Interestingly, these two genera are known to contain a set of bacterial genes for cellulose and xylan hydrolysis. In contrast, these bacteria were found to be decreases in the HFLP group (Fig. 2).

### Fatty acid profile of longissimus dorsi muscle

To determine the difference in fatty acid composition in *Longissimus dorsi* muscle of twin sheep fed different feed composition, gas chromatography-mass spectrometry analysis was utilized to establish their fatty acid profiles. A total of thirty different kinds of fatty acids were detected in the muscle samples. Of these, eighteen showed a significant difference between twins under different diets. The concentration of seven of these fatty acids was significantly lower in the muscle of sheep fed a HFLP diet compared with that of sheep fed an LFHP diet. Besides, the concentration of eleven fatty acids was higher in the muscle of sheep fed a HFLP diet (Table 3).

**Table 3:** Fatty acid profile of *longissimus dorsi* muscles of sheep fed two different diets

	HFLP	LFHP	*P* value^a^
Palmitic acid	23.58 ± 2.46	25.95 ± 3.22	0.0170
Oleic acid	43.23 ± 2.25	45.16 ± 2.31	0.0149
Linolenic acid	0.22 ± 0.02	0.39 ± 0.04	0.0000
Margaric acid	0.70 ± 0.06	0.82 ± 0.06	0.0000
Margaroleic acid	0.23 ± 0.04	0.31 ± 0.03	0.0002
Stearic acid b2	0.33 ± 0.04	0.37 ± 0.04	0.0002
Jecoleic acid	0.05 ± 0.02	0.07 ± 0.02	0.0286
Tridecanoic acid	0.01 ± 0.00	0.01 ± 0.00	0.0016
Myristic acid b	0.03 ± 0.01	0.02 ± 0.01	0.0002
Nonadecanoic acid	0.10 ± 0.02	0.09 ± 0.01	0.0472
Arachidonic acid	0.10 ± 0.03	0.08 ± 0.02	0.0225
Pentadecenoic acid b2	0.11 ± 0.03	0.09 ± 0.02	0.0014
Palmitic acid b	0.12 ± 0.02	0.10 ± 0.02	0.0008
Palmitelaidic acid t	0.24 ± 0.03	0.21 ± 0.03	0.0021
Arachidic acid	0.17 ± 0.03	0.11 ± 0.02	0.0003
Elaidic acid	0.46 ± 0.14	0.28 ± 0.11	0.0019
cis Linolelaidic acid	2.51 ± 0.38	2.12 ± 0.22	0.0002
Stearic acid	23.60 ± 3.83	19.62 ± 2.96	0.0101

^a^ A paired *t*-test was used to determine the significance pf the difference between the two groups

^b^ branched chain fatty acids

^t^ trans fatty acids

### Differential gene expression is observed in twins under different feed regimes

To compare the transcriptomes from twin sheep fed with two different feeds, we utilized an Illumina Hi-Seq 2500 to sequence eight *longissimus dorsi* muscle samples, and generated 22.5 to 28.5 million 125-bp clean paired-end reads. The Trinity software was used to reconstruct a reference transcriptome using obtained RNA-seq reads. This reference transcriptome contained 347,335 transcripts with an average length of 2166.29 bp. The expression values of transcripts of the eight samples were calculated by RSEM, whereas the edgeR package in R was used to analyze differentially expressed genes (DEGs) between twin lambs under different diets. In total, 487 differentially expressed transcripts corresponding to 368 DEGs were identified, including 67 novel genes, with *P =* 0.0001, and a false discovery rate (FDR) = 0.05 [16](Additional file 3). Of these genes, the PDK4 gene, which plays a key role in the regulation of glucose and fatty acid metabolism, had the highest expression levels in *longissimus dorsi* muscle samples.

**Fig. 3:**
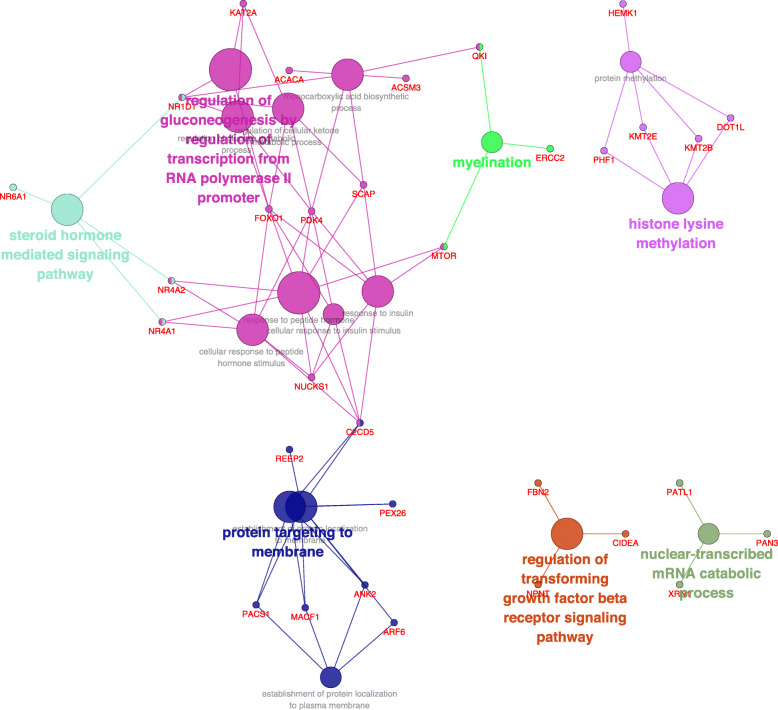
KEGG pathways enrichment analysis based on DEGs in muscles of sheep under diets. In this schematic representation of the pathways in which DEGs participate, node size shows the significance of a term, the bigger the node, the higher the significance of the term identified. The color of the nodes represent the proportion of genes from each cluster that are associated to each term

To inquire the function of identified DEGs, a Kyoto Encyclopedia of Genes and Genomes (KEGG) pathway enrichment analysis was performed using ClueGO. A total of 105 enriched pathways were identified. These pathways were classified into 22 clusters involved in several functions, such as epigenetic modification, muscle cell development, muscle fiber assembly, regulation of the glucose metabolic process, RNA transport, and the steroid hormone mediated signalling pathway (Fig. 3). With regard to epigenetic modification, KALRN, NTM1B and TET1 genes were highly expressed in the muscle of sheep fed with alfalfa pellets (LFHP diet), whereas DSRAD, DOT1L, KAT2A, KMT2B, KMT2E, HSF4, PHF1, and PICK1 genes were highly expressed in muscles of sheep fed with *Ceratoides* pellets (HFLP diet). These results showed that a HFLP diet could upregulate more genes related to epigenetic modification compared with a LFHP diet. Besides, results show that a HFLP diet may inactivate genes involved in steroid hormone mediated signalling pathway such as NR1D1, NR4A1, and NR4A2. Results also revealed that members of the steroid-thyroid hormone-retinoid receptor superfamily could build up a bridge between steroid hormone mediated signalling pathways and the regulation of glucose metabolism (Fig. 3).

### Metabolome differences between longissimus dorsi muscle samples of sheep twins are induced by feeds

**Fig. 4:**
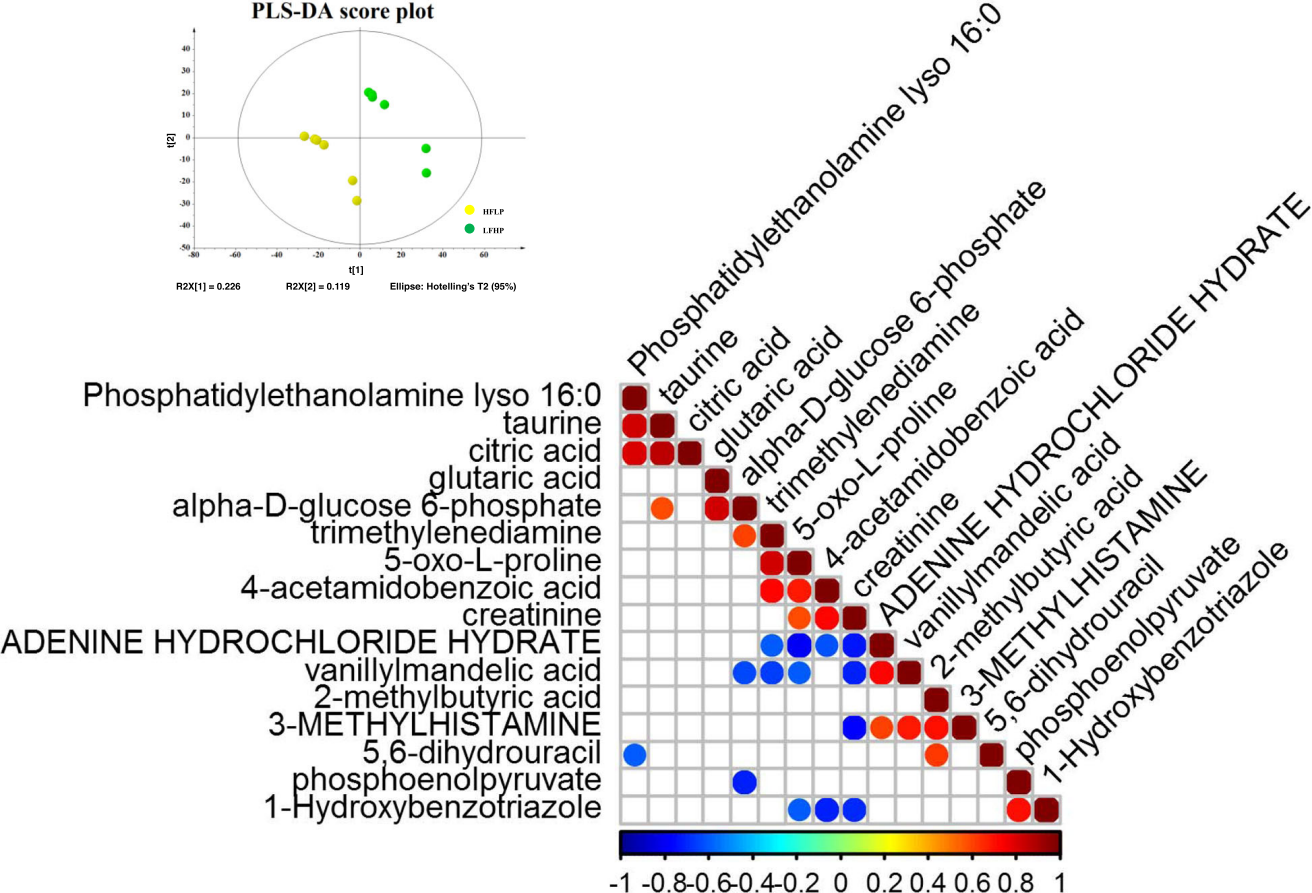
Clustering and correlation analyses of the muscle metabolome. **a**. Partial least squares discrimination analysis of the metabolome of muscle samples from sheep fed either an HFLP diet (yellow dots) of a LFHP diet (green dots). **b**. Metabolite-metabolite correlation analysis showing positive correlations (red dots) and negative (blue dots) correlations

To identify metabolic differences among twin sheep fed different diets, a partial least squares discriminant analysis (PLS-DA) was performed between the two groups (Fig. 4a) and sixteen compounds were identified, with VIP > 1.5, *P* < 0.05 and FDR < 0.05, as key for separating the two groups (Table 4). A Pearson correlation coefficient analysis was utilized to analyze the metabolite-metabolite correlation among identified metabolites (Fig. 4b). Most of them were involved in glucose metabolism, such as phosphatidylethanolamine, methylhistamine, phosphoenolpyruvate, alpha-D-glucose 6-phosphate, and taurine. Phosphoenolpyruvate and alpha-D-glucose 6-phosphate are involved in gluconeogenesis; both of them were found to be activated in muscle samples of the HFLP group. Furthermore, we observed a change in the concentration of methylhistamine, which is an inactive metabolite of histamine that plays a role in the activation of the histamine H2/H4 receptor. Besides, methylbutyric acid is related to the odor of the bioproduct, and adenine hydrochloride and taurine are essential factors for the differentiation and growth of skeletal muscles.

**Table 4:** Relevant compounds identified in the metabolome *of longissimus dorsi* muscle of fed LFHP OR HFLP diets

Compounds	VIP	***P*** value	FDR	log2FC (LFHP / HFLP)
Adenine hydrochloride hydrate	1.81	0.02	0.03	−3.28
Dihydrouracil	1.63	0.04	0.04	−1.30
Phosphoenolpyruvate	2.08	0.00	0.03	−1.24
Methylbutyric acid	1.74	0.04	0.04	−0.66
Methylhistamine	1.79	0.04	0.04	−0.48
Hydroxybenzotriazole	1.97	0.02	0.03	−0.46
Vanillylmandelic acid	1.98	0.02	0.03	−0.36
Proline	1.78	0.04	0.04	0.48
Phosphatidylethanolamine lyso 16:0	1.71	0.03	0.04	0.60
Creatinine	2.11	0.01	0.03	0.71
Alpha-d-glucose 6-phosphate	1.99	0.01	0.03	0.71
Trimethylenediamine	2.09	0.01	0.03	0.96
Glutaric acid	1.79	0.02	0.04	1.06
Citric acid	1.64	0.04	0.04	1.18
Taurine	2.00	0.01	0.03	1.81
Acetamidobenzoic acid	2.31	0.00	0.01	3.09

*FDR* Significances were considered at q < 0.05, *VIP* variable importance in the projection

### Integrated analysis of microbiota, transcriptome and metabolome

**Fig. 5:**
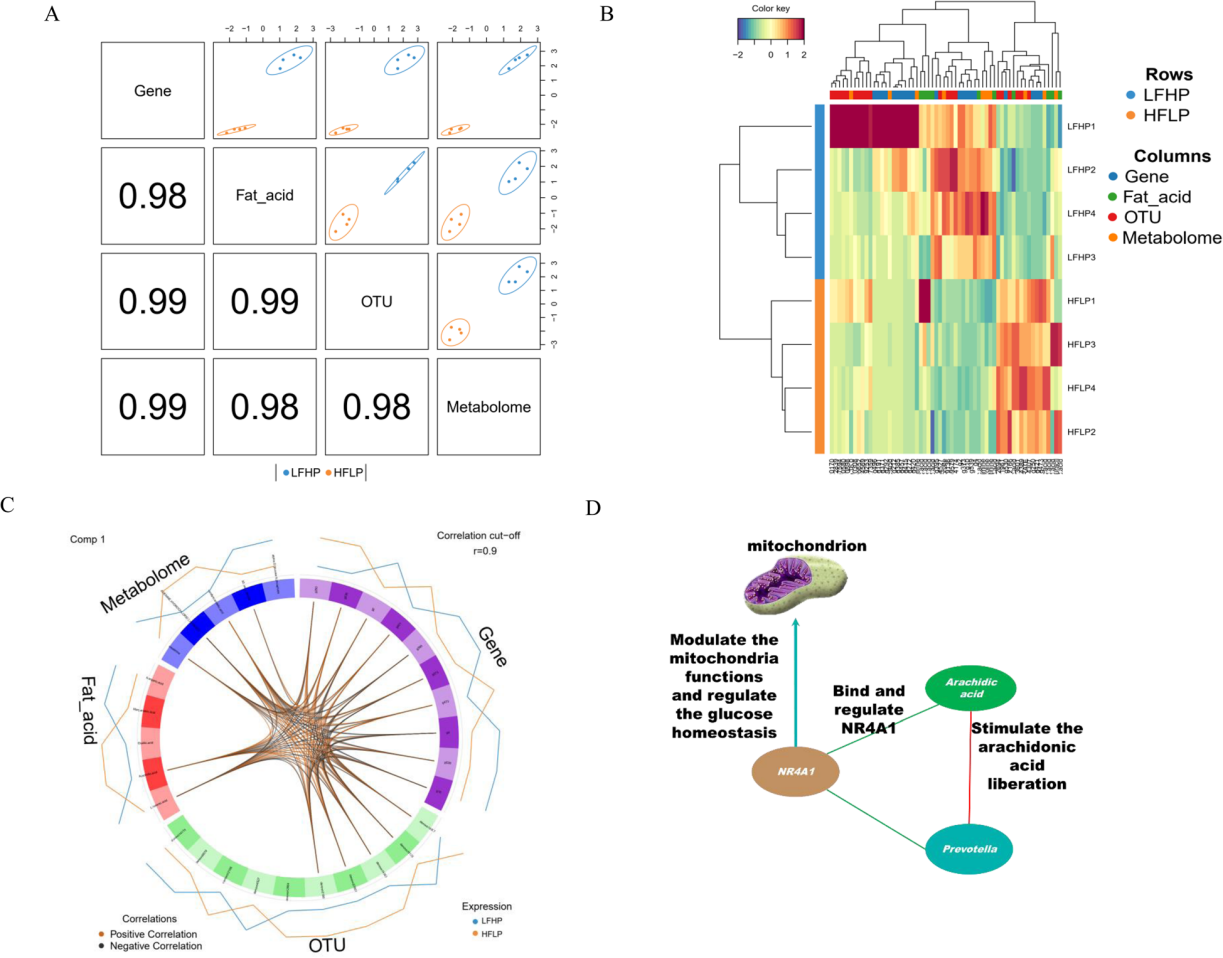
Multiple omics integrative analysis for the four kinds omics data obtained in this study. **a**. Sample scatterplot made with plotDiablo, displaying the first component in the four data set (upper diagonal plot) and the Pearson correlation between each component (lower diagonal plot). **b**. Clustered image map (Euclidean distance, Complete linkage) of the multi omics signature. Information from the eight sheep in the study are represented by rows, whereas selected features from each of the four omics datasets are represented by columns (according to the color code line below the cladogram). **c**. Circos plot showing positive (brown lines) and negative (black lines) correlations (r > 0.9) between selected features from each dataset (feature names appear in each quadrant). **d**. Potential cross talk between multiple molecular layers. Red line, positive correlation; green line, negative correlation; blue arrow

DIABLO software was utilized to integrate multiple omics data, including the rumen microbiome, and the transcriptome and metabolome of the muscles. The correlation between the components of each data set was maximized as specified in the design matrix. Results showed that, together with fatty acid profiles, all four kinds of data, representing different levels, had a high correlation at the component level (Fig. 5a). Based on the multi omics molecular signature expression for each sample, the eight samples were clustered into two classes, consistent with the feed group to which they belonged (Fig. 5b). A circos plot built using 5 OTUs (*Veillonellaceae, RF9 uncultured bacterium, Prevotellaceae, Lachnospiraceae and Rikenellaceae*), 10 genes (LOC105606886, RHDF2, NR4A1, CN093, PLCB3, GRHL1, NUCKS1, PPR3C, AL2CL and comp108473_c2_seq4), 2 fatty acids (linolenic and arachidic acid), and 4 metabolites (alpha-D-glucose 6-phosphate, vanillylmandelic acid, creatinine, and adenine hydrochloride hydrate) and a correlation coefficient threshold of 0.9, demonstrated notable divergences between the two diets (Fig. 5c). Among multi-omics molecular, a potential cross talk between multiple molecular layers was discovered (Fig. 5d).

## Discussion

The microbiota of the rumen is an intermediate link between diet and nutritients that are absorbed by sheep. In this study, feedstuff composition affected the microbiota structure of sheep rumen. The *Ceratoides* pellets increased the diversity of rumen microbiota compared to that seen in sheep feed with alfalfa pellets, consistent with a previous study in humans that showed that a high-fiber diet could increase the potentially beneficial microbiome [17]. In this study, we found that the major organisms in sheep rumen microbiota were members of the Bacteroidetes and Firmicutes phyla. The predominant phylum was Firmicutes, rather than Bacteroidetes in the proximal duodenum microbiota. Consistently, a marked decrease in the Bacteroidetes / Firmicutes ratio from the rumen to the colon was observed in cows [18]. This ratio also changed significantly in the rumen of sheep consuming either *Ceratoides* or alfalfa pellets (3.63 and 9.61 respectively). The Bacteroidetes / Firmicutes ratio is considered as an index of gut homeostasis [12], which also varies with age and weight [19, 20]. Different kinds of nutrition from diet intake have different effects on the Bacteroidetes/Firmicutes ratio. However results may vary from one species to another, e.g., a high-energy diet could increase the level of Firmicutes and decrease Bacteroidetes in humans [17] and mice, while the opposite trend was has been observed in the rumen of sheep. This reason may occur because sheep, humans, and mice have different digestion types [21]. The rumen microbiota has some special microbial species to help ruminants degrade feeds. In this study, the genus *Prevotella* showed to be at low abundance in the rumen of sheep under a HFLP diet, which is not consistent with a previous study in humans and pigs [22]. *Prevotella* is largely responsible for much of the proteolytic activity within the rumen and is important for breaking down plant proteins into usable nitrogen, which becomes accessible for other microbes within the rumen [23].

In this study, low quality feeds (HFLP) could not provide sufficient nutrition for sheep. After microbial fermentation in the rumen, metabolites could be absorbed by the host and regulate its physiology. As FOXO1 and PDK4 were highly expressed in the *longissimus dorsi* muscle of HFLP fed sheep. FOXO1 could up regulate the expression level of the PDK4 gene by directly binding to its promoter region. It is known that PDK4 plays an important role in maintaining normal blood glucose levels and increasing fat metabolism in response to prolonged fasting [24] and starvation [25]. Gene expression analysis also showed that the methylation process was down regulated in muscle samples of sheep fed with HFLP pellets. We observed that the heat shock factor protein 4 (HSF4) was highly expressed in the muscle of the HFLP group, which could be a response to low quality feed under the starvation stress. Furthermore, the interaction between HSF4 and FKBP5 plays a role in the intracellular trafficking of heterooligomeric forms of steroid hormone receptors maintaining the complex in the cytoplasm when unliganded. Moreover, when the steroid hormone-mediated signalling pathway is inactivated, both food uptake and the activity of a number of enzymes involved in hepatic TAG synthesis decreases [26]. This could be the reason for the reduced fat content found in muscle samples of the HFLP group.

Gluconeogenesis metabolites, which could maintain glucose levels in blood during starvation, were found to be activated in muscle tissues from the HFLP group, according to the metabolome analysis done. Besides, the content of methylhistamine in muscle of the HFLP group was higher than that in muscles of the LFHP group. Methylhistamine, which can stimulate gastric acid secretion [27] and induce an increase in the cAMP [28], is an agonist for the histamine H2/H4 receptor. With an increase in cAMP concentration, pyruvate kinase would be phosphorylated by protein kinase A and its activity would decrease [29] and the glycolysis process was inhibited in the HFLP muscle. Meanwhile, the taurine and the adenine hydrochloride were both upregulated in the LFHP group and could promote the differentiation and growth of muscles [30, 31].

We further utilized the Multiple-omics integrative analysis and identified glucose metabolism as the most significant pathway. 5 OTUs, 10 genes, 2 fatty acids and 4 metabolites assigned to this correlation demonstrated notable divergences between two diets. The potential cross-talk was found between four omics data layers. The *Prevotella* could stimulate the arachidonic acid liberation by the activity of phospholipase A2 [32]. The arachidonic acid could interact with the ligand-binding domain of the orphan nuclear receptor NR4A1 [33]. NR4A1 uses its transcriptional activity to modify mitochondrial proton leak, to modulate the mitochondria functions [34], and thereby to regulate the glucose homeostasis [35]. The glycolytic pathway was activated when the expression of NR4A1 reduced [36]. Taking together, the cross-talk could plays a role in the regulation of multiple molecular layers. However, it needs more advanced functional assay are needed to identify which is the most critical step in this network altering the content and quality of mutton.

As the rumen microbiota changes, the fatty acid profile and the metabolome of the *longissimus dorsi muscle* of twin sheep are also affected by the two different kinds of feeds. The integrated analysis showed that arachidic acid and linolenic acid were significantly correlated with the microbiota of the rumen, DEGs, and the metabolism of *longissimus dorsi muscle.* Arachidic acid and linolenic acid are both essential fatty acids for humans; thus, we could produce a functional mutton for consumers through sheep diet modification.

## Conclusions

Feed nutritional content may affect the microbiota structure of rumen and change the epigenetic regulation of genes involved in the glucose metabolic pathway. Low quality feeds induced gluconeogenesis to maintain glucose levels in blood, resulting in decreased fat content in the muscle. Multiple omics analysis showed that the structure of the microbiota was significantly correlated with the metabolome and gene expression in muscle. This study laid a theoretical foundation for controlling the nutrient intake of sheep, modifying its fatty acid spectrum, and removing any material that may be detrimental to the quality of mutton, which could direct sheep feeding for the obtention of functional mutton.

## Methods

### Pellet feed preparation

We prepared two kinds of pellet feeds, one consisted of 25% concentrate supplementation (Lamb Feed 585, Charoen Pokphand Group of Inner Mongolia, China) and 75% alfalfa hay, whereas the other consisted of 25% concentrate supplementation and 75% *C. arborescens* hay. Because of its higher protein content of alfalfa is the most prevalent forage legume for livestock worldwide. *C. arborescens* is a forage grass with a high fiber content, widely distributed in the north of China. *C. arborescens* pellets contained 57.2% fiber and 11.8% crude protein (high fiber low protein, HFLP, content), whereas alfalfa pellets contained 46.2% fiber and 16.1% crude protein (low fiber high protein, LFHP, content) (see Additional file 1).

### Animal experiments and sampling

The unique matching of twins provides researchers with ways to analyze complex traits together with approaches to understand how genes and the environment interact [37]. To decrease the influence of genetic background, four pairs of Chinese Sunit sheep (*Ovis aries*) female twins weighing 24 ± 2.3 kg were used in a matched pairs experiment, which were randomly divided into two groups and fed for 12 weeks either with HFLP and LFHP pellets. Sheep (*Ovis aries*) twins were selected from the sheep breeding farm of the Inner Mongolia Academy of Agricultural & Animal Husbandry Sciences located at Siziwangqi, Jining, China (N41.798°E111.858°). All experiments were performed under the guidance of the Institutional Animal Care and Use Committee (IACUC) in the Inner Mongolia Academy of Agricultural & Animal Husbandry Sciences, China. Both groups were kept in a small corral with free feeding.

After a fasting period, lambs were slaughtered in a local abattoir. The animals were euthanised via captive bolt stunning, followed by exsanguination. After slaughter, two pieces of the *longissimus dorsi* muscle between the 12th and 13th ribs were sampled from each individual and preserved in a nitrogen canister. Samples were frozen in a freezer at − 196 °C and − 80 °C and used for the extraction of total RNA and fatty acid profile and metabolome analysis, respectively.

For microbiome analysis, both the liquid and solid phases of rumen content were separated by squeezing them through four layers of gauze (1 mm mesh) (Additional file 2). The fluid was then centrifuged at 500 g for 30 min at 4 °C to isolate residual particles and preserved at − 80 °C until extraction of genomic DNA.

### Microbiome DNA preparation, 16S gene sequencing and processing

In this study, we sequenced the 16S rRNA gene of the microbiome in sheep rumen to identify the bacterial community structure associated with animals under two different diet conditions. Briefly, total DNA was isolated from the rumen’s liquid content and 16S rRNA genes were amplified for subsequent metagenomic sequencing using an Illumina MiSeq PE300 system (Illumina). Microbiome DNA was extracted using the E.Z.N.A.®Stool DNA Kit according to the manufacturer’s instructions. Bacterial 16S genes were amplified from microbiome DNA using the V3-V4 region primers and sequenced using the Illumina MiSeq PE300. After filtering and merging, 868,901 tags were acquired from the eight samples (an average of 108,613 tags per sample). Then, we used the uclust [38] algorithm in QIIME (version 1.8.0) [39] to cluster the thousands of identified tags with, at least, 97% similarity to obtain the operational taxonomic units (OTUs). The annotation of these OTUs was carried out using the Silva database [40]. The alpha and beta diversities were calculated using Mothur v.1.30 [41] and UniFrac method [42], respectively.

### Fatty acid profile and meat quality of the longissimus dorsi muscle

Carcass traits and meat quality of the *longissimus dorsi* muscle were measured. Crude fat was extracted from muscle tissues using hexane. Then, the sulfuric acid-methanol method was used, to prepare the ester derivatives of the fatty acids; they were then separated and identified by gas chromatography-mass spectrometry analysis (Agilent 6890-5973 N, USA). Gas chromatography was performed using a DB-23 quartz capillary column (60 m × 0.25 mm × 0.25 μm), using nitrogen as the carrier gas (ultra-high purity) at 1.0 mL/min,, the injector temperature was set to 270 °C, whereas the sample transfer line were set to 280 °C, the conditions were as follows: 130 °C start, 6.5 °C/min to 170 °C, 1.5 °C/min to 215 °C, 40 °C/min to 230 °C, and 3 min hold. Mass spectrometer conditions were set to record mass spectra in the electron ionization (70 eV), quad temperature 150 °C, Source temperature 230 °C,, and in full scan mode over the atomic mass range of 35-380 amu. A paired *t*-test of in R was used to determine the significance of the differences found between the two groups, differences were considered significant at *P* < 0.05.

### Metabolome of the longissimus dorsi muscle

In this study, metabolites were isolated from the *longissimus dorsi* muscle and analyzed by liquid chromatography followed by mass spectrometry (LC/MS). We performed un-targeted metabolomics analysis of the *longissimus dorsi* muscle obtained from the loin between the 12th and 13th ribs of four pairs of lamb twins. The identification of the components was based on the untargeted analysis workflow reviewed by (Patti, Yanes and Siuzdak). After data preprocessing, the differences between the two groups were determined by principal components analysis (PCA) and PLS-DA: *P* ≤ 0.05 + VIP ≥ 1. Aiming to increase sample size for our metabolomics analysis, we created two new samples by the mixing sample A with sample B to E (1:1, v/v), and sample C with sample D to F (1:1,v/v). In total, we detected 4013 ions, of which 2293 could be putatively assigned to metabolites according to five databases; the including Human Metabolome Database (HMDB) (http://www.hmdb.ca), Metlin (http://metlin.scripps.edu), massbank (http://www.massbank.jp/), LipidMaps (http://www.lipidmaps.org), and mzclound (https://www.mzcloud.org). To explain differences in the muscle tissue, we normalized the peak areas using autoscaling (centering, scaling, and transformations were done to improve the biological information content of our data).

### RNA library construction and sequencing

Total RNA was extracted from *longissimus dorsi* muscle samples with Trizol reagent according to the manufacturer’s protocol. We measure the concentration, integrity, and purity of the extracted RNA using a NanoDrop, spectrophotometer, a Qubit 2.0 and an Aglient 2100. An OD ratio (260/280) between 1.8 and 2 with RIN > 7 is generally accepted as a measure for good quality RNA that may be used for further experimentation. mRNAs were enriched with oligo (dT) magnetic beads. Then, we used fragmentation buffer to implement random fragmentation, useful as a template to synthesize cDNA with random hexamers, dNTP, RNase H and DNA polymerase I. cDNA was then purified, end repaired, and joined to the adapters. AMPure XP beads were used to carry out fragment size selection. Then, the following quality control analysis of the cDNA library was done. Sequencing was conducted on an Illumina HiSeq 2500 platform.

### Gene expression analysis of longissimus dorsi muscle

After filtering the raw *RNA-seq* data*,* between 5.43 GB to 6.92 GB clean bases were acquired for the eight muscle tissue samples used. We used the Trinity software to perform de novo transcriptomes assemblies [43]. Subsequently, assembly statistics were performed using TrinityStats.pl scripts. We calculated the gene expression value of reads per kilobase million (RPKM) for each muscle sample using RSEM software [44]. Significantly DEGs were determined by edgeR [45]. Multiplicity correction was performed by applying the Benjamini-Hochberg method on the *P* values, to control the false discovery rate (FDR) [46]. We did not run any qPCR for DEGs to validate the RNA-Seq technique because we focused on finding regulatory networks and pathways from the high-throughput data, which is always far better than the additional information obtained from confirming some few candidates [47, 48]. We performed an enrichment analysis of these differential genes with ClueGO [49] which is a plugin of Cytoscape [50].

### Multiple-omics integrative analysis

To integrate and explore the different kinds of omics dataset, DIABLO, a mixOmics R package was used [51]. For supervised integrative analysis of multiple omics, the classification performance of the final sparse PLS-DA model is assessed with the perf function by specifying a prediction distance. Performance is measured via overall misclassification error rate and Balanced Error Rate (BER) [51]. The muscle DEGs and the metabolome and fatty acids were analyzed together with the microbiome of the rumen searching for a highly correlated multi omics signature that could explain the different characteristics of mutton from sheep grown under two different diets (Additional file 4).

## Supplementary information

**Additional file 1.** Nutrition component of two feed stuffs.

**Additional file 2 **Contents of rumen of sheep feeding with the two kinds of pellets *Ceratoides* pellets (HFLP: A1-D1) and Alfalfa pellets (LFHP: A2-D2).

**Additional file 3.** Differentially expressed genes between muscle tissues of sheep fed HFLP and LFHP diets.

**Additional file 4.** Schematic representation of the multiple omics analysis made in this study.

## Abbreviations

BER: Balanced Error Rate
DEGs: Differential expression genes
FDR: False discovery rate
HFLP: High fiber low protein
KEGG: Kyoto encyclopedia of genes and genomes
LC/MS: Liquid chromatography / mass spectrometry
LFHP: Low fiber high protein
OTUs: Operational taxonomic units
PCA: Principal components analysis
PLS-DA: Partial least squares discriminant analysis
RPKM: Reads per kilobase million
VIP: Variable importance in the projection
