# Supplementary material for: Multiple omics analysis reveals that high fiber diets promote gluconeogenesis and inhibit glycolysis in muscle
Source: BMC Genomics. 2020 Sep 24;21:660. doi: 10.1186/s12864-020-07048-1 (PMC7513505; doi:10.1186/s12864-020-07048-1)
Supplement: Supplementary file 1 — Additional file 1. Nutrition component of two feed stuffs. [file 12864_2020_7048_MOESM1_ESM.docx]

Additional file 1 Nutrition component of two feed stuffs.

|  | DM (%) | GE (MJ/kg DM) | CP (% DM) | CF (% DM) | ADF (% DM) | NDF (% DM) |
| --- | --- | --- | --- | --- | --- | --- |
| LFHP | 89.4 | 16.3 | 16.1 | 2.4 | 25.2 | 46.2 |
| HFLP | 90.5 | 15.8 | 11.8 | 2.2 | 29.6 | 57.5 |

ADF, Acid Detergent Fiber; CF, Crude Fat; CP, Crude Protein; DM, Dry Matter; GE, Gross Energy; NDF, Neutral Detergent Fibre.
