# Supplementary material for: Multiple omics analysis reveals that high fiber diets promote gluconeogenesis and inhibit glycolysis in muscle
Source: BMC Genomics. 2020 Sep 24;21:660. doi: 10.1186/s12864-020-07048-1 (PMC7513505; doi:10.1186/s12864-020-07048-1)
Supplement: Supplementary file 2 — Additional file 2 Contents of rumen of sheep feeding with the two kinds of pellets Ceratoides pellets (HFLP: A1-D1) and Alfalfa pellets (LFHP: A2-D2). [file 12864_2020_7048_MOESM2_ESM.docx]

Additional file 2 Contents of rumen of sheep feeding with the two kinds of pellets *Ceratoides* pellets (HFLP: A1-D1) and Alfalfa pellets (LFHP: A2-D2).
