# Supplementary material for: Multiple omics analysis reveals that high fiber diets promote gluconeogenesis and inhibit glycolysis in muscle
Source: BMC Genomics. 2020 Sep 24;21:660. doi: 10.1186/s12864-020-07048-1 (PMC7513505; doi:10.1186/s12864-020-07048-1)
Supplement: Supplementary file 4 — Additional file 4. Schematic representation of the multiple omics analysis made in this study. [file 12864_2020_7048_MOESM4_ESM.docx]

Additional file 4 Schematic representation of the multiple omics analysis made in this study.
